# Formal mindfulness practice predicts reductions in PTSD symptom severity following a mindfulness-based intervention for women with co-occurring PTSD and substance use disorder

**DOI:** 10.1186/s13722-022-00333-2

**Published:** 2022-09-16

**Authors:** Vanessa C. Somohano, Josh Kaplan, Aurora G. Newman, Maya O’Neil, Travis Lovejoy

**Affiliations:** 1 Portland VA Medical Center, Mental Health and Neuroscience Division, Portland, OR 97239 United States; 2 Oregon Health and Science University, Portland, OR 97239 United States; 3 Pacific University, Hillsboro, OR 97123 United States

**Keywords:** Meditation, PTSD, Craving, Substance use disorder, Dual diagnosis, Women, Mindfulness-based interventions

## Abstract

**Background:**

Women with co-occurring posttraumatic stress disorder (PTSD) and substance use disorder (SUD) experience systemic barriers that place them in danger of poorer treatment outcomes. Some mindfulness-based interventions (MBIs) have demonstrated efficacy in reducing PTSD and SUD symptoms. Mindfulness practice is a core component of MBIs, thought to elicit and maintain positive behavioral change; however, no research to our knowledge has assessed the role of mindfulness practice on sustained treatment gains among women with co-occurring PTSD-SUD. Such research is necessary to better inform MBIs for dually diagnosed women.

**Methods:**

This secondary analysis assessed whether post-intervention formal and informal mindfulness practice predicted reductions in PTSD symptoms and substance craving 6 months following an 8-session mindfulness-based relapse prevention intervention for women diagnosed with co-occurring PTSD-SUD (*N* = 23). Data were derived from a pilot randomized controlled trial evaluating the feasibility and preliminary efficacy of a trauma-integrated mindfulness-based relapse prevention program for women with co-occurring PTSD-SUD.

**Results:**

Greater duration of formal mindfulness practice (i.e., minutes per practice) predicted reduced total PTSD symptoms ($\upbeta$ = − .670, *p* < .00), trauma-related avoidance ($\upbeta$ = − .564, *p* = .01), arousal and reactivity ($\upbeta$ = − .530, *p* = .02), and negative cognitions and mood ($\upbeta$ = − .780, *p* < .01) six months following treatment. Informal practice did not predict any outcomes.

**Conclusions:**

This research highlights the potential role of formal mindfulness practice in sustaining reductions in PTSD symptoms over time among women with co-occurring PTSD-SUD. Further study of strategies to promote ongoing formal mindfulness practice in this population following a MBI are warranted.

*Trial registration* The parent trial was registered with ClinicalTrials.gov (Identifier: NCT03505749).

Derived from ancient Buddhist traditions and secularized for Western medical contexts, mindfulness-based interventions (MBIs) have demonstrated efficacy across clinical populations and settings [1]. A core component of MBIs thought to foster processes that elicit change in mental health outcomes is mindfulness practice [2]. MBIs typically utilize formal and informal mindfulness practice to enhance the qualities of developing attention and concentration on present moment experiences and insights into causes and conditions of skillful and unskillful behaviors. Formal mindfulness practice is defined as a set time during which one engages in meditation exercises intended to foster compassion and nonjudgmental awareness to the present moment (e.g., body scan meditation, mindful movement) [3]. Informal mindfulness practice is defined as attending to daily tasks with present-focused awareness and equanimity (e.g., mindful eating) [3].

Formal mindfulness practices are beneficial for a variety of mental health outcomes, and have demonstrated efficacy in reducing depression, anxiety, and stress, and increasing emotion regulation, compassion, and quality of life [4, 5]. Mechanistic studies have found that the effect of formal practice on reduced stress, depression, and anxiety are explained by mindfully engaging in daily events [6]. Further, better formal practice quality has explained the relationship between time spent practicing and reductions in depression and anxiety [7]. Informal mindfulness practice has also been associated with improved mood, enhanced positive affective states, mindfulness, and quality of life [8, 9], and reduced perceived stress, depression, and anxiety [9].

MBIs have shown promise in reducing craving, severity of substance dependence, and relapse rates among a wide variety of substance use disorders (SUDs; [10]. MBIs have also shown acceptability among populations with SUD and heightened risk of trauma exposure (i.e., directly experiencing or witnessing a life threatening event or sexual violence) including justice-involved women [11], veterans [12], racial and ethnic minorities [13], and first responders [14]. Among trauma-exposed individuals who have also been diagnosed with Posttraumatic Stress Disorder (PTSD)—a psychiatric condition comprising four symptom clusters (i.e., intrusive thoughts, avoidance behavior, negative changes in cognitions and mood, changes in arousal and reactivity) following exposure to a life threatening event or sexual violence for at least 1 month following the incident—MBIs have demonstrated reductions in PTSD symptom severity and improved functional outcomes [15].

While extant literature on MBIs for SUDs is promising and has demonstrated superior longitudinal outcomes to active control and treatment-as-usual conditions [16], little is known about how formal and informal mindfulness practice impacts SUD. One study found that greater frequency and duration of formal mindfulness practice weakened the positive relationship between craving and substance among trauma-exposed individuals after participation in mindfulness-based relapse prevention [17]. Another study assessed enactment of formal and informal home practice on alcohol and drug use in a sample of individuals in SUD treatment and found that greater home practice predicted less substance use and craving at 2 and 4 months following participation in mindfulness-based relapse prevention [18]. No known studies have assessed the impact of formal and informal mindfulness practice on PTSD symptom severity despite available evidence of efficacy of MBIs among those diagnosed with PTSD [19].

Formal and informal mindfulness practice is designed to enhance qualities of mindfulness [20, 21]; thus, conceptualization of practice indices (e.g., frequency, duration, and quality) as mechanisms of change in clinical outcomes after participation in MBIs, rather than using subjective measures of trait mindfulness, is needed. Self-report measures of trait mindfulness have shown weak or nonexistent relationships with frequency and duration of mindfulness practice, and the use of self-report measures of mindfulness is consistently cited as a limitation of mindfulness-based intervention research [22]. Using behavioral indicators (i.e., frequency and duration of mindfulness practice) known to enhance mindfulness traits may be a more accurate reflection of mechanisms driving change within MBIs [20].

While some cross-sectional studies have assessed the effect of formal and informal mindfulness practice among trauma-exposed individuals with SUD following a MBI, no studies to date have assessed the impact of formal and informal mindfulness practice on longitudinal outcomes among women diagnosed with PTSD and co-occurring SUD. Given that women with SUD and PTSD may be particularly suited for MBIs [23], it is important to investigate whether key intervention targets such as formal and informal mindfulness practice predict reduced SUD and PTSD symptom severity, and clarify whether these targets inform MBIs for women with co-occurring PTSD-SUD. Moreover, assessing longitudinal treatment gains among dually diagnosed women is imperative as risk for relapse, psychiatric severity, and functional impairment is heightened compared to women with either disorder alone [24], particularly within the first 12-months after completing SUD treatment [25].

The current study is a secondary analysis of a pilot randomized-controlled trial that assessed whether formal and informal mindfulness practice during the course of a mindfulness-based relapse prevention intervention predicted reductions in PTSD symptomatology and substance craving 6-months post-intervention in a sample of women with co-occurring PTSD-SUD (*N* = 23). We hypothesized that greater duration of formal and greater frequency of informal mindfulness practice at post-course would predict reductions in craving and PTSD symptom severity 6-months following treatment.

## Method

### Participants

Participants in the parent study were recruited from residential and intensive outpatient SUD treatment clinics in the region of the United States by clinicians, fliers, and oral presentations given to patients and staff by research personnel. All participants were provided oral and written informed consent before screening procedures. Enrollment in the parent study required a current dual diagnosis of co-occurring PTSD-SUD and being self-identified as a woman. Overall, 96 participants were recruited and screened. Of those, 92 were eligible and 83 were enrolled in the parent study. Participants (*N* = 23) who had complete data for baseline, post-course, and 6-month follow-up measures were included in the current analyses. All procedures were approved by the associated Institutional Review Board.

A Mindfulness-Based Relapse Prevention (MBRP) [26] protocol was adapted for the parent trial and consisted of 8 60-min sessions over a 4-week period. Each session began with a guided formal or informal mindfulness practice, discussion of a weekly theme related to substance use (e.g., managing triggers) or PTSD, and ended with a formal or informal mindfulness practice learned during session. Mindfulness practices were led by MBRP-trained, graduate-level clinicians. Participants were provided with CDs and CD players during session 1 with recordings of mindfulness practices to be used during the intervention to facilitate home practice assignments. For more details of the protocol used in the parent study, see [26].

### Measures

PTSD symptom severity was assessed with the Posttraumatic Checklist for the DSM-5 (PCL-5; [27] a 20-item, self-report measure assessing PTSD symptoms in the last month. The scale is rated from 0 to 4 for each symptom, and individual items are summed to create a total PTSD symptom severity score, along with four PTSD symptom cluster subscale scores—intrusions, avoidance, negative changes in cognitions and mood, and changes in arousal and reactivity. Internal consistency of the PCL-5 for the current study was excellent (*α* = 0.91). Craving was measured with the adapted Penn Alcohol Craving Scale (PACS; [28], a 5-item, self-report measure assessing frequency, intensity, and duration of substance craving for the prior week. Each item is scaled from 0 to 6, where 0 = “Never” and 6 = “All the time.” Internal consistency for the current study was excellent (*α* = 0.93). Formal practice was measured by self-reported minutes of daily meditation practice, and informal practice was measured by self-reported frequency of daily engagement in mindfulness skills to everyday activities. Participants were assigned to record their daily formal and informal mindfulness practice using a homework log over the 4-week intervention period, which included practice completed during class. During the post-course assessment timepoint, participants reported their average daily formal and informal practice during the intervention period. An average duration of formal practice minutes per week and frequency of informal practice engagement per week were then calculated and used for the present analysis.

### Planned analyses

Data were missing completely at random (MCAR) [29]; thus, listwise deletion was employed, which is recommended for MCAR, and is known to produce unbiased estimates and conservative results [30]. Multiple linear regression analyses were used to assess whether formal and informal mindfulness practice during the intervention period predicted 6-month substance craving and PTSD symptom severity. PTSD symptom severity (model one) and craving (model two) were entered as dependent variables into two separate models. Formal and informal practice were entered separately as independent variables into both models. Baseline PTSD symptom severity was entered as a covariate for model one, and baseline craving was entered as a covariate for model two. We also conducted post-hoc analyses to assess whether formal and informal mindfulness practice predicted reductions in specific PTSD symptom clusters at 6-month follow-up. Bivariate correlations of average formal practice minutes and informal practice frequency during the course, and 6-month PTSD symptom severity and craving were conducted to confirm associations prior to conducting regression analyses. All inferential analyses used an alpha level of 0.05 and two-sided tests of significance. A post hoc power calculation was conducted to determine the adequacy of the present sample size. Given the effect sizes (Cohen's f^2^) observed in the regression models (PCL model: f^2^ = 0.66 [large]; PACS model: f^2^ = 0.21 [medium-large]), α = 0.05, and a sample size of *N* = 23, the present study achieved an average power of 0.67 (PCL model = 0.91; PACS model = 0.43).

## Results

### Demographics

Participants’ mean age was 36.13 years old (*SD* = 7.97, range = 24–57). Most participants self-identified as Non-Hispanic White (82.6%) and endorsed methamphetamines as a primary drug of choice (65.2%). See Table 1 for full sample characteristics and Table 2 for means, standard deviations, and correlations for dependent and independent variables, respectively.

**Table 1:** Sample characteristics of participants at baseline

Baseline characteristic	M	SD	Min	Max
Age	36.13	7.96	24	57
Age of first alcohol use	13.56	3.13	7	21
Age of first tobacco use	13.43	3.3	7	18
Age of first illicit substance use	15.78	4.3	8	26
Age of first trauma experience	8.28	7.23	0	26
Session attendance	5.56	2.44	0	8
Previous times in treatment	3.21	2.02	1	6

	*N*	%		
Race				
White	19	82.6		
Multiracial	2	8.7		
Does not identify with race listed	2	8.7		
Ethnicity				
Hispanic	4	17.4		
Non-Hispanic	19	82.6		
Sexual orientation				
Heterosexual	14	60.9		
Bisexual	9	39.1		
Primary substance of choice				
Methamphetamine	15	65.2		
Alcohol	6	26.1		
Heroin	1	4.3		
Prescription opiates	1	4.3		
Treatment setting				
Outpatient	13	56.5		
Inpatient	10	43.5		

*N* = 23

**Table 2:** Means, standard deviations, and correlation coefficients of study variables at baseline, postcourse and 6-month follow-up

Variable	Baseline		Postcourse		6 m Follow-up		Correlations			
	M	SD	M	SD	M	SD	1	2	3	4
1. Formal practice minutes	–	–	203.37	155.06	–	–	–			
2. Informal practice frequency	–	–	33.48	27.11	–	–	.147	–		
3. PTSD symptoms	47.39	11.81	29.61	12.57	31.08	17.20	− .469*	− .017	–	
4. Craving	22.30	7.84	9.42	7.84	6.95	6.24	− .394	.192	.496*	–

^*^p < .05, two tailed. Bivariate correlations of average formal practice minutes, average informal practice duration, 6-month PTSD symptom severity, and 6-month craving are displayed

### Primary analyses

Greater duration of formal mindfulness practice during the intervention period predicted reductions in PTSD symptom severity, *t*(21) = − 3.53, *p* < 0.01, $\beta$ = − 0.670, such that every minute increase in weekly formal mindfulness practice was associated with a 0.67-point reduction in PTSD symptoms at 6-month follow-up. Frequency of informal mindfulness practice during the intervention period did not predict reductions in total PTSD symptoms at 6-month follow-up, *t*(18) = − 0.12, *p* = 0.91, $\beta$ = − 0.033. Formal, *t*(21) = − 1.63, *p* = 0.12, $\beta$ = − 0.327, and informal mindfulness practice, *t*(18) = 1.03, *p* = 0.32, $\beta$ = 0.262, did not predict reductions in substance craving at 6-month follow-up. See Table 3 for regression coefficients.

**Table 3:** Regression coefficients of duration of formal and frequency of informal mindfulness practice during the course on 6-month PTSD symptom severity and craving

Variable	Model 1					Model 2				
	β	SE	95% CI	R^2^	∆R^2^	β	SE	95% CI	R^2^	∆R^2^
BL symptoms	.658**	.267	[.480, 1.16]	.602	.427	− .327	.166	[− .602, .009]	.262	.145
PC FMP	− .670**	.019	[− .120, − .039]			− .381	.009	[− .035, .003]		
BL symptoms	.106	.392	[− .687, .996]	.011	.001	− .314	.196	[− .660, .179]	.131	.065
PC IMP	− .033	.175	[− .379, .335]			.262	.063	[− .071, .220]		

*N* = 23. We examined the predictive strength of average formal and informal mindfulness practice during the course on total PTSD symptom severity and craving 6 months following the intervention. In Model 1, 6-month total PTSD symptoms is entered as the dependent variable. In Model 2, 6-month craving is entered as the dependent variable

*BL* baseline, *PC *postcourse, *FMP *formal mindfulness practice, *IMP *informal mindfulness practice

^**^p < .01

### Post-hoc analyses

Given the significant predictive relationship between duration of formal mindfulness practice during the course and PTSD symptom severity, post-hoc analyses were conducted to examine the relationship between formal practice and symptom clusters of PTSD. Greater duration of formal mindfulness practice during the intervention period predicted significant reductions in PTSD-related avoidance, *t*(21) = − 2.94, *p* = 0.01, $\beta$ = − 0.564, changes in arousal and reactivity, *t*(21) = − 2.55, *p* = 0.02, $\beta$ = − 0.530, and changes in negative cognitions and mood, *t*(21) = − 4.29, *p* < 0.01, $\beta$ = − 0.780, at 6-month follow-up. Formal mindfulness practice was not predictive of reductions in intrusion symptoms (*p* = 0.08) at 6-month follow-up.

## Discussion

To our knowledge, this is the first investigation of the role of duration of formal practice and frequency of informal mindfulness practice in predicting longitudinal changes in PTSD and SUD symptom severity in a sample of women with co-occurring PTSD-SUD. We hypothesized that greater duration of formal and frequency of informal mindfulness practice during a mindfulness-based intervention would predict reductions in craving and PTSD symptom severity 6-months following treatment. Our hypothesis was partially supported: greater duration of formal mindfulness practice minutes predicted reduced PTSD symptom severity. However, frequency of informal mindfulness practice did not predict reductions in PTSD symptom severity, and neither formal nor informal practice predicted reductions in substance craving. Post-hoc analyses were conducted to examine the relationship between duration of formal mindfulness practice and PTSD symptom clusters. These analyses revealed that duration of formal mindfulness practice minutes significantly predicted symptom reductions in avoidance, arousal and reactivity, and changes in negative mood and cognitions 6-months following treatment.

Formal mindfulness practice may impact PTSD symptoms via pathways similar to exposure-based strategies utilized in evidence-based interventions for PTSD such as prolonged exposure therapy. For example, meditation practitioners and prolonged exposure patients alike are taught to attend to arising and passing stimuli, including aversive trauma-related experiences, without engaging in avoidance behaviors (e.g., substance use) [31, 32]. This common practice between meditation and prolonged exposure may increase tolerance for experiencing negative affective states, enhance access to higher-order cognitive functioning to respond to aversive experiences more skillfully, and extinguish previously conditioned substance use behaviors when faced with trauma symptoms. Moreover, habituation to aversive experiences via approach-based coping fostered through formal mindfulness practice and trauma-focused therapies may reduce hyperarousal and numbing when confronted with PTSD symptoms. Indeed, MBIs have demonstrated reductions in biomarkers associated with PTSD-related arousal [33, 34].

Additionally, cultivation of nonjudgmental attitudes may mitigate negative thoughts and affective states associated with trauma. For example, nonjudgment has been shown to reduce shame [35], which is further associated with reductions in PTSD symptom severity [36]. Furthermore, approaching trauma-related thoughts with a non-judgmental stance may help an individual de-identify with such thoughts, which in turn may reduce their salience. Indeed, meditation practices taught within MBIs specifically target thoughts and encourage an individual to observe their transient nature rather than identifying with their content. Mindfulness practice has consistently demonstrated increases in emotional well-being [3], which may also reduce PTSD-related affect and cognitions. Given that PTSD symptoms are associated with substance use [37], increasing tolerance to aversive experiences and optimizing one’s repertoire of skills to mitigate symptoms may attenuate the relationship between PTSD and SUD.

Our post-hoc finding that duration of formal practice was not predictive of craving at 6-month follow-up was counterintuitive, and contradicts previous findings that mindfulness training reduces substance craving compared to control conditions [38]. It is possible that formal mindfulness practice does not account for a significant amount of improvement in substance use craving. Studies demonstrating the efficacy of MBIs for craving may highlight other elements of mindfulness training responsible for improvement such as quality of mindfulness practice, reductions in negative affect, increases in emotion regulation and emotional well-being, and group cohesiveness [15, 39]. Our results support research suggesting that MBIs may be a viable treatment approach for targeting PTSD-related avoidance, arousal, affect, and cognitions among women in SUD treatment [23], and provide evidence for the efficacy of trauma-integrated MBIs for SUD. Moreover, engagement in formal mindfulness practices throughout the duration of an MBI appears to be safe and optimizes longitudinal treatment gains among dually diagnosed women; however, more research with a larger sample size is warranted.

## Limitations

This study has several limitations. First, the sample for the parent study was recruited from a relatively small and homogenous geographical area. This may limit the generalizability of our findings to other more heterogeneous regions and cultures. As the current study had limited power to detect the observed effect magnitude for alcohol craving (*b* = 0.43), findings would need to be replicated in larger samples to add greater confidence to the reliability of results observed in the current study. Second, outcome data were collected using self-report measures, which can be susceptible to issues such as practice effects and response bias [40]. Future research should seek to overcome these limitations by including objective measures (e.g., clinician-administered PTSD scales; objective assessment of mindfulness practice frequency and duration) and by recruiting from larger and more diverse pools of patients. Last, data on other group factors (e.g., cohesion, treatment engagement, therapist characteristics) were unavailable in the current study but should be included as covariates in future research assessing the impact of mindfulness practice on longitudinal treatment gains following a MBI.

## Conclusions

Despite these limitations, the findings of this study present implications for the utility of mindfulness in the treatment of PTSD symptom severity among women with co-occurring PTSD and SUD. Our findings also extend the literature by identifying specific elements of MBIs (e.g., greater duration of formal mindfulness practice) that may be of particular benefit for certain PTSD symptom clusters (e.g., avoidance, arousal, negative changes in mood or cognition).

## Data Availability

The datasets generated and/or analyzed during the current study are not publicly available due to unavailable platform for encrypted data sharing, but are available from the corresponding author on reasonable request.

## Abbreviations

MBIs: Mindfulness-based interventions
PTSD: Posttraumatic stress disorder
SUD: Substance use disorder
